# Urban public health education services, health status, and increased fertility intentions of the rural migrant population

**DOI:** 10.1186/s12978-023-01648-2

**Published:** 2023-07-24

**Authors:** Yanshuo Huang, Long Miao, Bei Lyu

**Affiliations:** 1 Business School, Xiangtan University, Xiangtan, China; 2 School of Economic, Hunan University of Finance and Economics, Changsha, China; 3 School of Economics and Management, Huaibei Normal University, No. 100, Dongshan Road, Xiangshan District, Huaibei, 235000 Anhui Province China; 4 Chinese Graduate School, Panyapiwat Institute of Management, Nonthaburi, Thailand

**Keywords:** Urban public health education services, Health status, The rural migrant population, Fertility intentions, Standard of living

## Abstract

**Purpose:**

Health education services in urban public health represent a significant guarantee to improve health status, reduce fertility pressure, and uplift the living standard of the rural migrant population.

**Methods:**

Based on the data from the 2018 China Mobility Monitoring Survey, this research paper analyzes the association between urban public health education services and the fertility intentions of the rural migrant populations.

**Results:**

The study findings indicate that (i) the education services in urban public health demonstrate a significant positive effect on the increase in fertility intentions of the rural migrant population; (ii) further, improvement in the health status represents a crucial mechanism by which urban public health’s education services influence the fertility intentions; (iii) in addition, the education services of urban public health exert a significant impact on improvement in the fertility intentions through public health consultation, promotional materials, SMS services, and face-to-face consultation; (iv) finally, urban public health’s education services exhibit a significant influence on improvement in the fertility intentions of the rural migrant population with firm residence intention and low work intensity.

**Conclusions:**

This study extends empirical evidence for the government authorities to formulate policies to consummate the urban public health service system, strengthen the efficiency of urban public health education services, and improve the fertility intentions and the living standards of the rural migrant populations.

## Introduction

Fertility has always been an imperative concern in China, whereas fertility policy, as a significant means of fertility regulation, has often been assigned various connotations at different times in China. Meanwhile, the “family planning” policy enacted in 1982 not only effectively alleviated the population pressure on resources and the environment but also promoted social progress and economic development. Although parallel to this, it is pertinent to mention that in the past 40 years, China’s fertility rate has lowered to 1.3 in 2020 from 2.86 in 1982, which is extensively below the required level of 2.1 to maintain population generation replacement; thereby, ringing the alarm of the “low fertility trap” [26]. In order to cope with the impending population aging crisis, China has responded instantly and formulated policy adjustments, ranging from the “one-child policy” in 2013, and the comprehensive two-child policy in 2016, to the implementation of the “three-child policy” in 2021. Nevertheless, there is still an unsatisfactory effect of fertility promotion. Consistent with this, the “low fertility trap hypothesis” attempts to explain the phenomenon of low fertility and narrates that the reasons behind the low fertility intention of the current population of childbearing age chiefly lie in the reduction of the women’s inertia of childbearing age triggered by the fertility policy implementation, the imbalance between the consumption concept of the offspring and the income level, and the inter-generational transmission of fertility concepts [26]. Simultaneously, certain researchers argue that owing to social and economic development, the fertility intentions of the childbearing age population may also be affected by different factors such as living standard, external work pressure, and fertility cost pressure [2, 6, 41]. Therefore, the formulation of fertility policies based on the factors that affect the fertility intentions of the childbearing age population may serve as an effective way to both raise the fertility rates and avoid the potential “low fertility trap”.

The fertility intentions analysis of the childbearing age population highlights that the rural migrant population, as a huge collective formed by the “wave of urbanization”, deserves special attention from researchers and academicians across the world. Owing to the rapid development of China’s economy, the construction and development of the cities constitute a significant demand for labor. Since cities demonstrate better living resources (including health and education) as compared to the rural areas, therefore, there is a large flow of rural people to cities. For instance, when considering public health education services, both rural and urban public health education services belong to the national basic public health service system. There is no significant difference in the general content, but there are obvious differences in the method and quality of service. In terms of education methods, urban public health education service exhibits more diversified publicity methods (electronic information, professional consultation, and expert lectures), while rural public health education service is majorly based on volunteer lectures and publicity materials. In terms of resource investment, the funding and sponsorship for education services in rural public health are much less than that for education services in urban public health. Simultaneously, urban public health education services also possess more professionally-educated service personnel and a complete set of supporting facilities.

In the face of the temptation of rich resources in city life, more and more rural populations are moving to cities. However, how to alleviate the stress of rural migrants in their new urban lives has become a new problem. The seventh national census data states that the rural outflow population of China is nearly 272 million from 2010 to 2020. Nonetheless, there is a need to not underestimate the pressure of life faced by rural migrants in cities. In accordance with some reliable statistics, on the one hand, there are around 27 billion square meters of commercial housing in the urban regions, with a social asset value of nearly 300 trillion Yuan; on the other hand, there are about 22 billion square meters of housing in the rural areas, with an asset volume of approximately 20 trillion Yuan. Although the overall region difference is insignificant, but the difference in terms of the total amount of assets is greater than 10 times.

Under the dual effect of urban living pressure and traditional fertility intention in rural China, rural migrant population has become the population that needs to be paid attention to in the study of fertility intention of Chinese population. From the perspective of “interruption theory”, the rural migrant population might be affected by both rural and urban concepts of fertility [40]. In addition, the rural migrant population is subject to substantially higher fertility pressure from the external survival environment as compared to the local population in their regions [29]. Therefore, it is of vital significance to focus on the fertility intentions of the rural migrant population in order to improve not only the living standard of the rural migrant population but also China's total fertility rates; thus, supporting the balanced population development. Besides this, the health management, lifestyle, and fertility philosophy of the rural migrant population retain specific rural attributes, whereas the environment and rhythm of urban life differ significantly from those of rural communities. As a result of this, health education services in urban public health services represent a critical channel to assist the rural migrant population to enhance their quality of life, rapidly integrate into the urban environment, strengthen their health, and establish appropriate fertility philosophy. Additionally, the rural migrant population, as the developers and builders of the city, should yield the benefits of urban development. However, certain studies reveal that it is difficult for the rural migrant population to access urban public services, as compared with urban residents. On the one hand, the rural migrant population (particularly, the inter-provincial migrant population) may encounter obstacles in accessing urban public health education services when these migrants do not possess local household since the public health services at present are essentially linked to household registration and there is a need to optimize the existing mechanism of transferring regional household registration in various provinces and municipalities of China [43]; on the other hand, since the rural migrant population demonstrates a lower income level and weaker health awareness, therefore this population lacks the initiative to pay sufficient attention and accept education services in urban public health education [46]. In addition, the limited capacity of public health services in China’s cities makes the rural migrant population more inclined to be “crowded out” when accessing public services [18]; consequently, posing a greater challenge to the fertility pressure of the rural migrant population. In order to extend proper social security for the rural migrant population of childbearing age, the “Health China 2030” plan asserts that there is a need to undertake a good job of equalizing basic public health and family planning services for the migrant population while focusing on the women of childbearing age, adolescents, and the migrant population by carrying out education and intervention on sexual morality, sexual safety, and sexual health. Meanwhile, the 19th National Congress Report suggests accelerating the development of equalizing public health services; hence, putting forward higher requirements for fundamental public services, and medical- and health service systems.

China attaches great significance to the rural migrant population’s access to health education services in urban public health and fertility intentions, though the academic research is still lagging. Hence, there is a dire need to determine the influence of access to urban public health education services on the fertility intentions of the rural migrant population. At the same time, there is also a need to ascertain the specific mechanisms and pathways of the proposed influence. To the best knowledge of the authors, these research queries have still not been precisely addressed in academic circles. Based on this, this research paper adopts the data from the 2018 China Mobile Population Dynamics Monitoring Survey (CMDS), in order to investigate the impact of urban public health education services on the fertility intentions of rural mobile populations, while exploring and confirming the critical influence mechanisms related to the improvement in the residents’ health status, and further performing a systematic analysis of the heterogeneous influence of health education’s delivery methods and rural mobile population groups. Thus, this research article extends a factual basis to promote the development of homogeneous health services in urban public health and the implementation of fertility policies.

## Brief literature review and theoretical hypothesis

### Research on fertility intentions

Fertility intentions refer to the demand-oriented and ideal preference of the person to produce children, which is based on multiple dimensions such as a planned number of children, a childbearing schedule, an ideal number of children, and gender preference for childbearing. In addition, Fertility intention is closely associated with actual fertility behavior, which represents a critical factor that affects the fertility rate of a region and even a country. Similarly, the living standard of residents and the level of regional economic development also significantly determine fertility intention. For instance, Malthus argued in “Principle of Population” (1798) that the process of rapid social and economic development in any region or economy is followed by a high willingness to produce children, which eventually leads to a population growth rate that excessively exceeds the rate of economic development; thus, leading to the “overpopulation trap” [39]. Although, the reality is opposite to the theory proposed by Malthus. In the historical process of industrialization, population agglomeration to urban areas forms economies of scale and unified large markets, global economic growth lifts a wave of social urbanization, and metropolis form shaped population and economic resources in space polarization, thereby, exerting a significantly inhibiting effect on fertility. As a result of this, urbanization and low fertility rate gradually develop a dynamic docking relationship [4, 20, 27, 28, 45]. Since the Industrial Revolution, both emerging and developed nations have witnessed the phenomenon of “low fertility”, with fertility rates below the population replacement level. Consequently, accelerated adverse population growth has led to the deepening of aging in a large number of countries. Driven by economic laws, “low fertility” demonstrates a trend of globalization [16]. Parallel to this, “Classical demographic transition theory” advocates that in the process of continuous urbanization and industrialization, the attributes of population replacement shall change to “low birth rate, low death rate, and low growth rate” from “high birth rate, high death rate, and low growth rate”; hence, finally maintaining the generation replacement level of 2.1 for a long period [13]. However, the fertility rate of more than half of the countries has dropped below the line in nearly a decade. Therefore, the maintenance of an equilibrium level for the long term has become difficult to achieve. Simultaneously, the low fertility crisis is no longer only in large cities that lead to industrialized societies. Similarly, the low level of economic development in emerging economies also gradually propagates low fertility. Based on this, the “Classical demographic transition theory” noticeably is no longer able to explain the development trend of modern society. Therefore, the researchers also put forward the “second demographic transition theory”. Accordingly, the proposed theory believes that modernization processes such as industrialization and urbanization represent a dual phenomenon. On the one hand, these processes shape the modern layout of industrial structure and urban population; while, on the other hand, these processes reconstruct the concepts of marriage, family, and fertility [23]. Besides this, the postmodern ideology has abandoned the traditional family concept of male superiority over females. Meanwhile, the formal requirements for the gender and number of children [31] have gradually faded, whereas “having many children” and “raising children for old age” are no longer only the expectations of the young people’s new generation to have and raise children [11, 25]. Several research scholars argue that health status has become a significant factor that influences the fertility intentions of the childbearing age population in the modern era, which can be reflected in both physical and psychological factors. On the one hand, individuals have become more susceptible to emotional infection from multiple media due to the in-depth application of information technology [1, 32]. In the meantime, the Internet conveys certain post-childbearing life and changes in physical health, exaggerated fear of childbearing, and other relevant information to the population of childbearing age. Resultantly, the population of childbearing age demonstrates significant psychological fear of childbearing [17, 36]. On the other hand, the work unhealthy living habits, and pressure stimulated by the increasingly accelerated pace of life, bring a remarkable physical burden to the population of childbearing age [21]. Furthermore, the expectation of “eugenic birth” makes the population of childbearing age hesitate when faced with the choice of childbirth under the state of declining physical quality.

### Research on public health services and fertility intentions of the rural migrant population

From the perspective of the main factors influencing fertility intention, this paper targeted the rural migrant population. Since the flow from the rural areas to a new city, in this part of the crowd for the birth of physical and mental health represents more substantial fluctuation, therefore the migrant population is compared in terms of the different situations faced by them such as social-, economic-, cultural-, and health pressure [34]. Consequently, the migrant population in the reproductive choice needs to further consider more factors. Simultaneously, the ideologies between rural and urban areas and the collision of different living environments complicate the lifestyles and fertility concepts of mobile populations [24]. Moreover, public health services, as an important tool to not only alleviate the pressure on residents’ life but also enhance the social integration of the mobile population [7], present an effective source to relieve the pressure on the rural mobile population to reproduce children, increase their willingness to have children, and guarantee the sound health status of the rural mobile population. Nonetheless, the specific influence of public services on fertility intention is still controversial. In the context of pension pressure, several scholars advocate that the improvement and optimization of public services shall lower pension pressure, and weaken the role of “raising children for old age”; resultantly, leading in a decline in the fertility rate. For instance, Cigno and Rosati [9] performed a study on Italian pensions, and concluded that the total fertility rate would decrease by 0.02, with each 10% increase in the per capita pension amount in Italy, similarly, Boldrin et al. [3], analyzed the social security in Germany and inferred that an incline in the level of social security in the nations, with relatively well-developed social security systems, will not only relieve pension pressure but also reduce fertility. Additionally, other scholars suggest that imperfect public services shall lead to a higher household consumption burden, whereas the improvement in medical and health security shall support the rise in the fertility rate [44]. In the same vein, certain studies integrated the aforementioned two viewpoints; and accordingly, implied that the economic level might moderate the effect of medical services and public health on fertility intention. In particular, medical services and public health may discourage fertility intention for families with comparatively high economic levels by relieving the pressure of supporting their elderly. Conversely, public health and medical services may reduce the family’s economic burden on health and medical treatment for families with relatively low economic levels; thus, enhancing their willingness to bear children [19]. Besides, Wang and Peng [42] examined the fertility intention of rural women and confirmed that the satisfaction of rural women with public health service guarantees represents a vital factor to improve their fertility intentions. Based on this, public health care services are highly significant to the rural migrant population. Though, a practical concern that has to be analyzed is that access to public health services may be difficult for the mobile population to a certain extent; further, the crowding-out effect of unequal provision of public health services [5] considerably enhance the health stress and economic burden of the migratory population; hence, lowering their fertility intentions [38]. In short, present research studies majorly focus on public health services and fertility intention. However, few scholars only directly investigate the effect of health education services in urban public health on the fertility intention of the rural migrant population. Particularly, the equalization of public health services has been gradually promoted since the launch of the National Basic Public Health Service project in 2009; thereafter, access to public health services has also continuously improved the sense of social integration, people contentment, and satisfaction of the rural migrant population. Thus, this research paper argues that education services in urban public health, as a critical element of urban public health services, directly impact the fertility intentions of rural migrant populations in the context of their quality and reputation. Furthermore, consistent with the aforementioned analysis, health status (including mental and physical health) has become a significant factor that determines the fertility intention of childbearing-age persons in the modern era. Hence, there is a need to determine whether urban public health education exerts an influence on the health status of the population. Further, this study also ascertains whether “reproductive health and maternal and child health education services” and “mental health services”, represent prominent tools to improve the physical quality of the childbearing age population and alleviate the “fear of childbearing” among the sub-components of health education services in urban public health. To sum up, this article postulates the following research hypotheses:

- H1: Education services in urban public health positively affect the increase in fertility intentions of the mobile population;
- H2: The improvement in physical health serves as a critical channel for urban public health education services to influence the fertility intention of the migrant population;
- H3: The alleviation of psychological pressure represents an imperative way for education services in urban public health to impact the fertility intention of the migrant population.

## Research design

### Data sources

The empirical data in this research paper is adapted from the China Migrants Dynamic Survey (CMDS) in 2018. The proposed survey was organized and implemented by the National Health Commission; thereby, covering information related to the migrant population’s fertility intention, health, residence intention, public health services, and employment. In addition, this survey is scientific, authoritative, large-scale, and targeted, which is entirely in the line with research domain of this research study. Consistent with this, this paper focuses on the fertility intentions of the rural migrant population. Based on the Chinese characteristics of the samples chosen in this paper, China has effectively opened the “two-child” and “three-child” policies, in order to promote fertility; consequently, there is a constant increase in the age range of individuals with fertility intentions. Simultaneously, an under 50 age is typically the period of career rise and consolidation; further, this period is also a time when the rural migrant population is faced with the dual pressure to choose between “starting a career” and “bearing children” in the city. Consistently, the sample of the rural migrant population in this research paper is targeted as the married migrant population of childbearing age between 15 and 50 years old who not only possess rural household registration but also have resided locally for more than half a year, which carries vital research value. Finally, this study presents a valid sample of 90,279 after sample screening.

### Variable selection and description

*Dependent variables* The fertility intention is the dependent/explained variable in this research paper. In accordance with the research question, “Do you have a plan to have children this year and subsequent year?” the binary variable of fertility intention is established, and the value is assigned to 1 in case the interviewee responds “yes” and 0 when the interviewee responds “had no idea”, “no”, or demonstrates a missing value.

*Independent variables* The urban public health education service is the independent/explanatory variable in this study. Accordingly, the variables of receiving urban public health education services are set based on the research question presented in the study questionnaire, “Have you received health education in the following areas in your present community/unit in the past year?” In the questionnaire, the value of 1 is assigned when the interviewees have received urban public health education services in the areas of “prevention and treatment of infectious diseases”, “prevention and treatment of occupational diseases”, “prevention and treatment of chronic diseases”, “reproductive health and maternal and child health”, “mental health”, “self-help in public emergencies”, and “other aspects”. Conversely, the value is 0 when the respondent has not participated in any of the health education.

*Covariates* This study empirically controls for a series of covariates, including individual, family, spouse, and mobility status attributes of the rural migrant population. Among them, individual characteristics control for age, education level, employment status, marital status, political status, the number of children, and participation in medical insurance; spouse characteristics control for the age difference between spouses and the education level of the spouse; family characteristics control for family income level, family ethnicity, and family housing burden; mobility status control for the length of residence in the inflow, range of population movement, and the effect of the provincial region of the entry place.

*Mechanism variables* The theoretical part implies that education services in urban public health indirectly impact their health status whereas directly affect the fertility intentions of the rural migrant population. Additionally, the variables of health status are classified into mental health and physical health. While subjective self-rated health is commonly employed to reflect the social and psychological health status of interviewees [12, 30]. Contrary to this, objective physical health directly reflects the respondents’ physical status. Meanwhile, mental health, in accordance with the questionnaire question state, “how do you think is your health status?” Reportedly, the interviewees respond to “health” with a value of 1 and “basic health” or “unhealthy” with a value of 0. Subsequently, physical health based on the research question stands “did you have any illness (injury) or physical discomfort in the past year?” When the interviewees respond “No”, the value stands 1. In contrast, when the participant answers “yes, the last time happened two weeks ago” and “yes, the last time happened two weeks ago”, the value is assigned to 0.

The Specific Definitions and Descriptive Statistics of Each significant Variable are Presented in Table 1.

**Table 1:** Definition of main variables and descriptive statistics

Variables		Symbols	Definition Method	Mean	Std
Dependent variable	Fertility intentions	FI	Whether the interviewee Plans to have Children in the Next Two Years: Yes = 1, No = 0	0.057	0.231
Independent variable	Urban public health education service	UPHES	Has the Interviewee Received at Least One Health Education: Yes = 1, No = 0	0.819	0.385
Covariates	Age	Age	Interviewee’s Age (Years)	35.740	7.329
	Education level	Edu	Interviewees’ Education: High School/Secondary School and Above = 1, Otherwise = 0	0.355	0.479
	Political identity	Party_member	Whether the Interviewee is a Member of the Communist Party: Yes = 1, No = 0	0.038	0.192
	Marital status	Marital	Marital Status of the Interviewee: First Marriage = 1, Remarried = 0	0.973	0.161
	Employment status	Work	Did the Interviewee Do More than One Hour of Paid Work in the Week before May Day: Yes = 1, No = 0	0.853	0.354
	Urban medical insurance	Insurance	Whether the Interviewee Participates in Urban Residents’ Medical Insurance, Urban Employees’ Medical Insurance, or Publicly Funded Medical Care: Yes = 1, No = 0	1.006	0.319
	Number of children	Child	Number of Biological Children of the Interviewee	1.495	0.700
	Spouse's education level	Spo_edu	Interviewee’s Spouse’s Education: High School/Junior High School and Above = 1, Otherwise = 0	0.359	0.480
	The age gap between couples	Age_gap	The Age Difference between the Interviewee’s Spouse and Interviewee	− 0.227	0.290
	Family ethnic identity	Ethnic	Whether Either Interviewee or Spouse is an Ethnic Minority: Yes = 1, No = 0	0.109	0.311
	Family income level	Income	Natural Logarithm of the Average Monthly Gross Income of the Interviewee’s Household	8.834	0.500
	Family housing stress	House_ exp	Interviewee Households’ Average Local Monthly Housing Expenditure to Total Expenditure Ratio (%)	0.122	0.153
	Range of population movement	Range_move	Whether the Interviewee is Inter-Provincial Mobility: Yes = 1, No = 0	0.498	0.500
	Length of residence in the inflow	Time_move	Interviewees have Lived in the Place of Inflow for a Long Time (Years)	6.262	5.577
Mechanism variables	Subjective level mental health	Mental health	Subjective Health Evaluation of Interviewees: Healthy = 1, Basically Healthy = 0, and Unhealthy = 0	0.894	0.308
	Objective level physical health	Physical health	Has the Interviewee been Sick (injured) or Unwell within One Year: Yes = 0, No = 1	0.896	0.305

### Empirical regression model setting

*Baseline regression model setting* With reference to relevant literature [22], the Probit model is applied to analyze the effect of urban public health education services on the fertility intention of the rural floating population, since the dependent variable was binary. The mathematical expression of the proposed model is as follows:

1 $$\Pr (FI_{i} = 1) = \alpha_{0} + \alpha_{1} UPHES_{i} + \alpha_{2} Control + \varepsilon_{i}$$

where $FI_{i}$ stands for the fertility intention of the rural migrant population, $UPHES_{i}$ indicates the urban public health education services, and $Control_{i}$ represents the set of control variables including spouse, family, individual, and mobility attributes of the rural migrant population. Lastly, $\varepsilon_{i}$ shows a random disturbance term.

*Estimation method selection and optimization* The rural migrant population does not demonstrate random access to education services in urban public health; further, it depends to a certain extent on their self-selection for the demand for urban public health education service. Consequently, there may exist selection bias in the estimation results when anticipating the impact of urban public health education services on the fertility intention of the rural migrant population. Parallelly, the propensity score matching method (PSM) is more generally used in academic circles to approximate a randomized trial [37]. Though, certain scholars hold that PSM carries substantial drawbacks, such as low accuracy of matching scores and poor covariate balancing when dealing with high-dimensional data. Contrarily, the entropic balanced matching method presents the benefits of being more robust, flexible, and balanced as compared to the PSM [14].

In this paper, the basic idea of the entropic equilibrium matching method is demonstrated below:

Primarily, binary dummy variables $UPHES_{i} = \left\{ {0,1} \right\}$ are constructed for rural migrant populations that attempt to access health education services in urban public health; where $UPHES_{i} = 0$ and $UPHES_{i} = 1$ signify the control- and treatment groups, respectively. Furthermore, $j$ covariates are introduced and afterward, the matrix $X_{i} = \left\{ {X_{i1} ,X_{i2} ,X_{i3} , \cdots \cdots ,X_{ij} } \right\}$ is constructed in this study. Consequently, the expression for sample means treatment effect is as follows:

2 $$ATT = E\left[ {Y_{1} |UPHES = 1} \right] - E\left[ {Y_{0} |UPHES = 1} \right]$$

In Eq. (2), $E\left[ {Y_{0} |UPHES = 1} \right]$ highlights the counterfactual result. Meanwhile, the entropic equilibrium treatment method directly scales the weights from the potential large equilibrium constraint set, and the relevant counterfactual estimation can be expressed as follows:

3 $$E\left[ {Y_{0} |UPHES = 1} \right] = \frac{{\sum {\left\{ {i|D = 0} \right\}Y_{i} w_{i} } }}{{\sum {\left\{ {i|D = 0} \right\}w_{i} } }}$$

In Eq. (3), $w_{i}$ connotes the entropy equilibrium weights chosen by the control group. Subsequently, these weights are adopted through the following re-weighting scheme, which majorly minimizes the entropy distance metric as follows:

4 $$\mathop {\min }\limits_{{w_{i} }} H\left( w \right) = \sum\limits_{{\left\{ {i|D = 0} \right\}}} {w_{i} \log (w_{i} /q_{i} )}$$

Equation (4) conforms to both the normality- and averageness constraints as follows:

5 $$\sum\limits_{{\left\{ {i|D = 0} \right\}}} {w_{i} c_{ri} \left( {X_{i} } \right) = m_{r} ,r \in 1, \cdots \cdots R}$$

6 $$\sum\limits_{{\left\{ {i|D = 0} \right\}}} {w_{i} = 1}$$

7 $$w_{i} \ge 0,\left\{ {i|D = 0} \right\}$$

where $q_{i}$ implies the benchmark weight and $c_{ri} \left( {X_{i} } \right) = m_{r}$ presents a set of R-balance constraints that are imposed on the covariate moments of the reweighted control group. Primarily, this step weights the covariates in the scheme while imposing constraints on each covariate with reference to Eq. (5). Consistent with this, the constraints include first-order moments (mean), second-order moments (variance), and third-order moments (skewness); thereby, intending to confirm that the covariate distribution moments between the reweighted treatment group and control group extend a consistent state.

In accordance with the above equilibrium constraint, non-negativity constraint, and normative constraint, the entropy equilibrium approach minimizes the entropy distance between $W = \left\{ {w_{i} , \cdots \cdots ,w_{{n_{o} }} } \right\}^{T}$ and the benchmark weight vector $Q = \left\{ {q_{i} , \cdots \cdots ,q_{{n_{o} }} } \right\}^{T}$ in Eq. (4) through searching and incorporating a set of unit weights $W$ [42]; and finally utilizing the proposed weights to carry out a weighted least squares regression to examine the impact of urban public health education services on the fertility intentions of the rural migrant residents.

*Endogeneity problem* Though the entropic equilibrium matching method effectively weakens the selectivity bias, this study may still represents endogeneity issue such as reverse causality and omission of important variables. Accordingly, the instrumental variables method is employed to resolve the possible endogeneity concern. Meanwhile, health education services in urban public health represent a crucial service component of urban public health services. Since public health services in China are extended through public bidding by county-level (district and city) governments to the society, and the winning organizations offer services to residents in their jurisdictions, therefore their funding majorly comes from the local-, provincial-, and central governments. Resultantly, the extent of local governments' corruption or anti-corruption practices may directly influence the quality and implementation status of urban public health education services. Based on this, the selection of the anti-corruption strength of local authorities as the instrumental variable of urban public health education services meets the required requirement. Simultaneously, the amount of corruption- or anti-corruption efforts of local government barely affects the fertility intentions of residents. In this study, the anti-corruption strength of local government in 2016 is adopted as the instrumental variable, which is still significantly associated with education services in urban public health in 2018. Parallel to this, it is more difficult to affect residents' willingness to bear children in 2018; thereby, satisfying the exclusivity requirements of the instrumental variable to a larger extent. Based on the aforementioned analysis, this paper employs the number of corruption and bribery cases filed per 10,000 public officials in 2016 to estimate the anti-corruption efforts in each province. Further, this estimation is used as an instrumental variable for the access to urban public health education services in rural migrant towns in 2018. Particularly, the data for measurement of the instrumental variables are obtained from the China Procuratorial Yearbook, the China Statistical Yearbook, and the work reports of provincial and municipal people’s procuratorates.

## Analysis of empirical results

### Analysis of baseline regression results

Firstly, Probit regression is adopted to explore the influence of urban public health education services on the fertility intention of the rural migrant population. Accordingly, Table 2 Columns (1), (2), and (3) all display the regression results of the Probit model, where (1) indicates the regression result without controlling for the province effects and control variables; (2) presents the regression results with controlling for the control variables but without controlling for the province effects. Furthermore, the baseline regressions in this study are chiefly based on Table 2 (3). In addition, the logit model is employed to further verify the robustness of the Probit model regression analysis, as depicted in Column (4). The results highlight that the choice to receive urban health education services effectively increases the fertility intention of the rural migrant population by 4.6%, which is significant at the 10% level of statistical significance. In terms of whether this study controls for control variables, province effects, or replaces the empirical regression model, the empirical results point out that receipt of urban public health education services exhibits a significant positive promotion influence on fertility intention, while the regression coefficients do not show significant variation; thus, confirming the hypothesis H1 of this study.

**Table 2:** Baseline regression and robustness tests

Variables	(1)	(2)	(3)	(4)
	Probit	Probit	Probit	Logit
UPHES	0.105***	0.058**	0.046*	0.107**
	(0.018)	(0.023)	(0.024)	(0.047)
Age		− 0.060***	− 0.060***	− 0.119***
		(0.002)	(0.002)	(0.003)
Edu		− 0.034	− 0.037*	− 0.053
		(0.022)	(0.022)	(0.043)
Party_member		− 0.000	0.006	0.008
		(0.039)	(0.039)	(0.074)
Work		− 0.454***	− 0.480***	− 0.960***
		(0.021)	(0.021)	(0.040)
Marital		− 0.551***	− 0.568***	− 1.042***
		(0.048)	(0.048)	(0.096)
Insurance		0.075***	0.063**	0.130***
		(0.026)	(0.026)	(0.050)
Child		− 1.039***	− 1.086***	− 2.116***
		(0.016)	(0.016)	(0.031)
Spo_edu		0.040*	0.031	0.055
		(0.021)	(0.022)	(0.043)
Age_gap		− 0.029	− 0.019	− 0.030
		(0.029)	(0.029)	(0.057)
Ethnic		0.119***	0.077***	0.105*
		(0.026)	(0.028)	(0.054)
Income		0.090***	0.070***	0.155***
		(0.018)	(0.019)	(0.037)
House_exp		0.062	0.078	0.112
		(0.058)	(0.059)	(0.119)
Range_move		− 0.095***	− 0.110***	− 0.226***
		(0.017)	(0.021)	(0.041)
Time_move		0.002	0.004*	0.008*
		(0.002)	(0.002)	(0.004)
Provincial effect	No	No	Yes	Yes
Constant	− 1.670***	1.465***	1.610***	3.131***
	(0.017)	(0.174)	(0.195)	(0.381)
Observations	90,279	90,279	90,279	90,279

① Values in parentheses represent robust standard deviations; ② ***, **, and * denote 1%, 5%, and 10% significance levels, respectively, as in the later section

The results of the control variables in Table 2 posit that, on the one hand, the level of household income and the length of residence in the migrant place significantly contribute to the fertility intentions of the rural mobile population. Based on this, the higher the family income level, the more willing the rural migrant population is to give birth, which is highly realistic. Moreover, the economic pressure brought by birth always has been a major concern that young people must consider when making birth decisions. However, it is not difficult to understand the impact of the residence length in the migrant place on the rural migrant population’s willingness to bear children. In general, a longer mobility time also implies a stronger intention to stay at a particular location. Owing to the growth of mobility time, the rural migrant population becomes more familiar with unfamiliar environments. Besides this, the amount of integration and adaptation to the environment often represents a crucial factor that influences the long-term settlement and child-rearing intentions of the migrants. On the other hand, inter-provincial migration, work status, first marriage, the age gap between the married partners, and the number of existing children exert a significant inhibiting impact on the fertility intention of the rural migrant population. The suppressive effects of work, the number of existing children, and the age difference between married partners are more significant on the fertility intentions of the rural migrant population, therefore it shall not be repeated again. Additionally, the inhibitory influence of interprovincial mobility on the fertility intentions of the rural migrant population is based on the fact that interprovincial mobility not only exhibits a more extended mobility range but is also prone to a more significant cultural gap. The proposed phenomenon is the same as the affecting mechanism of the residence length in the aforementioned migrant place. This means that the sense of integration and belonging are the important factors that influence the fertility intention of the rural migrant population. Besides, the first marriage negatively associates with fertility intentions among the rural migrant population, which may be unexpected for several individuals. On the one hand, in the context of sample data, half of the first-marriage population is under 35 years old, which indicates a crucial period for career advancement. The rural migrant population may display a higher pursuit of career during this period as compared to the non-migrant population. Therefore, more of the rural migrant population may prefer career achievement in terms of the priority between career and childbearing; On the other hand, the roles and emotions of the members are more complex than in the first marriage, regardless of whether the remarried parties bear children or multiple children. Further, the remarried families of childbearing age are often more willing to establish a closer and more stable relationship through childbirth, In order to prevent the re-fission of marriage and “raise children” and other considerations.

### Analysis of entropy equilibrium matching results

The “self-selection” of the rural migrant population in acquiring urban public health education services, owing to the impact of individual covariate attributes may lead to biased estimation results. Therefore, this paper incorporates the entropic equilibrium matching method to establish constraints on the first-order moments (mean), second-order moments (variance), and third-order moments (Skewness) on the covariates involved in the estimation process, to ensure that the control and treatment groups of the sample exhibit equal weighted means on each covariate and achieve exact matching of the sample, to determine whether to receive urban public health education on the differences in fertility intentions of the rural migrant population, in order to alleviate the problem of between-group bias in the effect assessment. Parallel to this, the matching results of entropy equalization in this paper are populated in Table 3. Specifically, the mean value, variance, and matching test results of each covariable are estimated prior to and after entropy equalization treatment. Reportedly, there exist relatively significant differences in the means and variances of the covariates prior to matching. Notably, the differences between the variances and means of the covariates of the two sample groups are significantly reduced after treatment. Furthermore, the standardized mean difference (SMD) between the control and treatment groups prior to and after entropy equalization is further estimated and the standard deviation T-test is performed, whereas the P-value of the standard deviation t-test is significantly 1 or highly convergent to 1; thereby, indicating that the covariates of the sample treatment and control groups are precisely matched after the entropy equalization treatment.

**Table 3:** Matching Test for Main Covariates

Variables		Mean		Variance		SMD	P-value
		Processing group	Control group	Processing group	Control group		
Age	Before matching	35.56	36.59	52.97	57.89	− 0.000071	0.000000
	After matching	35.56	35.56	52.97	52.97	0.000000	1
Edu	Before matching	0.374	0.2722	0.2341	0.1981	0.001598	0.000000
	After matching	0.374	0.374	0.2341	0.2341	0.000000	1
Party_member	Before matching	0.03981	0.03155	0.03823	0.03056	0.000794	0.000001
	After matching	0.03981	0.03981	0.03823	0.03823	0.000000	1
Work	Before matching	0.8585	0.8318	0.1215	0.1399	0.000808	0.000932
	After matching	0.8585	0.8585	0.1215	0.1215	0.000000	1
Marital	Before matching	0.9741	0.9696	0.02525	0.02952	0.000655	0.601395
	After matching	0.9741	0.9741	0.02525	0.02525	0.000000	1
Insurance	Before matching	1.011	0.9805	0.09868	0.1165	0.001136	0.000855
	After matching	1.011	1.011	0.09868	0.09868	0.000000	1
Child	Before matching	1.487	1.577	0.5136	0.5673	− 0.000644	0.000000
	After matching	1.487	1.487	0.5136	0.5136	0.000000	1
Spo_edu	Before matching	0.3737	0.2933	0.2341	0.2073	0.001262	0.000000
	After matching	0.3737	0.3737	0.2341	0.2341	0.000000	1
Age_gap	Before matching	− 0.2269	− 0.2252	0.08394	0.08391	− 0.000074	0.000000
	After matching	− 0.2269	− 0.2269	0.08394	0.08394	0.000000	1
Ethnic	Before matching	0.1069	0.1146	0.09549	0.1015	− 0.000296	0.000000
	After matching	0.1069	0.1069	0.09549	0.09549	0.000000	1
Income	Before matching	8.833	8.839	0.2675	0.3218	− 0.000082	0.000000
	After matching	8.833	8.833	0.2675	0.2676	0.000000	1
House_ exp	Before matching	0.2189	0.2014	0.04181	0.03751	0.001538	0.000000
	After matching	0.2189	0.2189	0.04181	0.04181	0.000000	1
Range_move	Before matching	0.4778	0.5754	0.2495	0.2443	− 0.001438	0.000000
	After matching	0.4778	0.4778	0.2495	0.2495	0.000000	1
Time_move	Before matching	6.196	6.755	30.83	38.72	− 0.000067	0.000000
	After matching	6.196	6.197	30.83	30.83	0.000000	1

$SMD = \left( {\overline{{X_{T} }} - \overline{{X_{C} }} } \right)/\sqrt {S_{T}^{2} \left( {n_{T} - 1} \right) + S_{C}^{2} \left( {n_{C} - 1} \right)/\left( {n_{T} + n_{C} - 2} \right)}$, where $\overline{{X_{T} }}$ and $\overline{{X_{C} }}$ indicate the means of each variable in the treatment and control groups, respectively, additionally, $S_{T}^{2}$ and $S_{C}^{2}$ represent the variances of each variable in the treatment and control groups, respectively, while $n_{T}$ and $n_{C}$ highlight the sample size in the treatment and control groups, respectively

Table 4 depicts the regression results of the fertility intentions of the rural migrant population after receipt of urban public health education services prior to and after entropy equilibrium matching. In order to further examine which items in urban public health education services exert a positive effect on the fertility intentions of the rural migrant population, this paper consists of the following research questions in the study questionnaire: “Have you received health education on the prevention and treatment of the occupational disease?”, “Have you received health education on reproductive health and maternal and child health?”, “Have you received health education on the prevention and treatment of infectious diseases?”, “Have you received health education on the prevention and treatment of chronic diseases?”, “Have you received health education on self-help in public emergencies?”, “Have you received health education on mental health?”, and “Have you received health education on other aspects?” This helps to represent the variables of health education manners while introducing different manners of health education into the model regression analysis. Meanwhile, the results propose that receiving “health education on mental health” and “health education on reproductive health and maternal and child health” exert a positive influence on the fertility intention of the rural migrant population. Since this result is highly close to the expectation of this study, therefore subsequently the endogeneity problem is tested in this paper.

**Table 4:** Comparison of the effects of urban public health education services on fertility intention before and after entropic equilibrium matching

Variables	Coefficient of public health service variables		Confidence interval at the 95% Level	
	Before processing	After treatment	Before processing	After treatment
Have received at least 1 health education	0.046*	0.030**	[− 0.001, 0.093]	[0.004, 0.056]
	(0.024)	(0.013)		
Occupational disease	− 0.149***	− 0.159***	[− 0.192, − 0.107]	[− 0.201, 0.117]
	(0.022)	(0.021)		
Infectious disease	− 0.069***	− 0.077***	[− 0.112, − 0.025]	[− 0.119, − 0.034]
	(0.022)	(0.022)		
Reproductive health and maternal and child health	0.382***	0.345***	[0.344, 0.420]	[0.311, 0.380]
	(0.019)	(0.017)		
Chronic disease	− 0.048**	− 0.051**	[− 0.095, − 0.002]	[− 0.097, − 0.005]
	(0.024)	(0.024)		
Mental health	0.052**	0.056**	[0.002, 0.101]	[0.007, 0.105]
	(0.025)	(0.025)		
Self-help in public emergencies	− 0.059***	− 0.069***	[− 0.098, − 0.019]	[− 0.108, − 0.030]
	(0.020)	(0.020)		
Other aspects	− 0.008	− 0.035	[− 0.057, 0.041]	[− 0.082, 0.012]
	(0.025)	(0.024)		

*** *P* < 0.01, ** *P* < 0.05, * *P* < 0.1

### Instrumental variable method regression

Though the entropy equilibrium matching method is capable to weaken the selective bias to a large extent, the IV-Probit model is employed in this research to alleviate endogeneity issues, such as mutual causation and missing variables, which may still exist in this research paper. From the perspective of first-stage regression, the regional anti-corruption efforts are significantly positive for the rural migrant population’s access to urban public health education services. This implies that the stronger the regional anti-corruption efforts, the higher the probability of the rural migrant population’s access to urban public health education services, which is consistent with the realistic logic. In addition, the F-value of the first stage joint test stands at 101.69; thereby, passing the test of a weak instrumental variable. Similarly, the Wald test P-value is recorded to be 0.015; hence, rejecting the hypothesis that the urban public health education service variable serves as an exogenous explanatory variable at the 5% level of statistical significance. This also suggests that there exists an endogeneity issue in this study whereas the derived results are biased and inconsistent due to sole dependence on the Probit model with the entropy equilibrium matching method. As a result, the results produced using the IV-Probit model shall be closer to the net effect. Meanwhile, IV-Probit regression results put forward that the rural migrant population's access to urban public health education services significantly enhances their fertility intention, which supports the Probit model and entropy equilibrium matching estimations. Based on this, the robustness of H1 is further confirmed in this research (Table 5).

**Table 5:** Instrumental Variable Method

Variables	(1)	(2)
	The first stage	The second stage
Anti-corruption	0.105***	
	(0.006)	
UPHES		0.930**
		(0.362)
Control variable	Yes	Yes
Provincial effect	Yes	Yes
The F-value	101.69	
Adj R-sq	0.0436	
Wald test P-value		0.015
Observations	90,583	90,583

*** *P* < 0.01, ** *P* < 0.05, * *P* < 0.1

### Mechanism test

The below mechanism test model is constructed for empirical analysis, in order to further explore the influential mechanism of the rural migrant population’s access to urban public health education services on their fertility intentions while verifying the existence of the theoretical transmission channel of health status in the previous study.

8 $$Health_{i} = \alpha_{0} + \alpha_{1} UPHES_{i} + \alpha_{2} Control + \varepsilon_{1}$$

9 $$FI_{i} = \gamma_{0} + \gamma_{1} Health_{i} + \gamma_{2} UPHES_{i} + \gamma_{3} Control + \varepsilon_{2}$$

where Health denotes the mechanism variable, this article selects the mental- and physical health status to describe consistent with the theoretical analysis. Consequently, the specific test steps are as follows: firstly, based on the Model (8), this test ascertains whether there exists a positive promotional effect association between urban public health education services and health status. Afterward, based on the significantly positive coefficient, health status is introduced into the basic empirical Model (1) to obtain Model (9), and by evaluating whether its coefficient and significance pass the mediation mechanism test.

Table 6 populates the regression results of the mechanism test, where Column (1) and (2) depicts the results of the mechanism test for mental health. Consequently, the results illustrate that the rural migrant population’s access to urban public health education services effectively improves their mental health status. Once the mental health status variable is integrated into the underlying empirical model, the coefficient of the mental health status on the fertility intention of the rural migrant population is positive, although this coefficient is not significant. Likewise, the coefficient of 0.040 for access to urban public health education services on the rural migrant’s fertility intention decreases, as compared to the baseline regression result of 0.046; thus, passing the mediation effect test to a certain extent. This infers that the access to urban public health education services by the rural migrants reports a facilitating effect on their fertility intentions by supporting their mental health status at the subjective level, though the influence of this mechanism is relatively weak. In terms of Columns (3) and (4), the results of the mechanism test for physiological health indicate that access to education services in urban public health by the rural migrant population effectively improves their physiological health. Once the physiological health status variable is introduced into the basic empirical model, the impact of physiological health status on the fertility intention of the rural migrants remains significantly positive. Consequently, access to education services in urban public health significantly influences the fertility intention of the rural migrant population. In particular, there is a decrease in the coefficient of 0.041 on the fertility intention of the rural migrant population, as compared to the baseline regression result of 0.046; resultantly, passing the mediating effect test. In short, improvement in the health status serves as a significant transmission mechanism that impacts the fertility intention of the rural migrant population when these migrants access urban public health education services. Furthermore, the transmission mechanism becomes more significant due to the improvement in the physical health status than the improved mental health status. Accordingly, the previous hypotheses H2 and H3 are verified in this study.

**Table 6:** Mechanism test—health status

Variables	(1)	(2)	(3)	(4)
	Mental health	FI	Physical health	FI
Mental health		0.023		
		(0.032)		
Physical health				0.057**
				(0.029)
UPHES	0.184***	0.040*	0.127***	0.041*
	(0.015)	(0.024)	(0.015)	(0.024)
Control variable	Yes	Yes	Yes	Yes
Provincial effect	Yes	Yes	Yes	Yes
Constant	0.679***	1.591***	1.022***	1.559***
	(0.133)	(0.197)	(0.132)	(0.197)
Observations	90,279	90,279	90,279	90,279

*** *P* < 0.01, ** *P* < 0.05, * *P* < 0.1

### Heterogeneity analysis

The above studies confirm that access to urban public health education services significantly influences the fertility intentions of the rural migrant population while verifying the transmission mechanism of health status. Additionally, there is a need to consider the following heterogeneity matters: on the one hand, there exist heterogeneous differences in the impacts of urban public health education services performed in different manners; on the other hand, there are also differences in terms of the degree of demand for and access to education services in urban public health by various groups of the rural migrant population. Hence, this paper shall analyze the heterogeneity of urban public health education services in urban areas in the context of differences in how the proposed education services are offered and the differences in the attributes of rural migrant population groups.

A heterogeneity analysis is carried out to determine how urban public health education services are delivered in towns and cities. This research study is based on the questionnaire question, “Did you receive the above health education activities in the form of promotional materials in your current community/workplace?”, “Did you accept the above health education activities in the way of health knowledge lectures in your current community/unit?”, “Did you receive the above health education activities as a publicity column in your current community/workplace?”, “Did you receive the above health education activities by SMS in your current community/workplace?”, “Did you receive the above health education activities in the form of public health consultation activities in your current community/workplace?”, “Did you receive the above health education activities in other ways in your current community/unit of residence?”, and “Did you receive the above health education activities in the form of individualized face-to-face consultation in your current community/workplace?” In addition to this, the methods of urban public health education services are categorized into seven types namely: “health knowledge lecture, public health consultation, SMS, promotional materials, face-to-face consultation, publicity column, and other ways”, while variables are defined respectively. Consequently, the value is assigned to 1 when the interviewees respond “yes”. Conversely, when the respondents respond “no”, the value is assigned to 0. In order to perform the way to the heterogeneity of regression results as illustrated in Table 7, based on the results, the promotional materials, SMS, public health consultation, and face-to-face counseling services in urban public health education effectively support the rural migrant population’s fertility intentions; thereby, also implying that a few kinds of urban public health education services are more readily accepted by the rural migrant population. Noticeably, this result is not difficult to understand. Most young people are not highly willing to spend time participating in lectures in the present relatively fast-paced urban life, specifically when the quality of lectures is not high; therefore it is convenient to make people tedious or even counterproductive; and the publicity column has long ceased to be a significant channel for residents to obtain information due to the transformation of information technology and modern lifestyle, therefore there is minimal effect of urban public health education services in the form of publicity column for young generation.

**Table 7:** Heterogeneity analysis–How UPHES is performed

Variables	(1)	(2)	(3)	(4)	(5)	(6)	(7)
Health knowledge lectures	− 0.009						
	(0.018)						
Promotional materials		0.048***					
		(0.018)					
Publicity column			− 0.002				
			(0.017)				
Public health consultation activities				0.051**			
				(0.022)			
SMS					0.060***		
					(0.018)		
Face-to-face consultation						0.109***	
						(0.026)	
Other ways							0.012
							(0.024)
Control variable	Yes	Yes	Yes	Yes	Yes	Yes	Yes
Provincial effect	Yes	Yes	Yes	Yes	Yes	Yes	Yes
Constant	1.651***	1.628***	1.649***	1.646***	1.643***	1.641***	1.647***
	(0.194)	(0.194)	(0.194)	(0.194)	(0.194)	(0.194)	(0.194)
Observations	90,279	90,279	90,279	90,279	90,279	90,279	90,279

*** *P* < 0.01, ** *P* < 0.05, * *P* < 0.1

Subsequently, a heterogeneity analysis is conducted concerning the differential characteristics of the rural migrant population groups. In this paper, the authors explore the potential heterogeneous effects of urban public health education services on the fertility intentions of rural migrant groups with different characteristics based on three perspectives: work intensity, literacy, and willingness to stay. The results of the heterogeneity analysis are presented in Table 8. Since the Probit model cannot directly compare group coefficients, therefore this article constructs interaction terms to identify the heterogeneity of different groups. Primarily Column (1) of Table 8 highlights the results of the heterogeneity analysis of work intensity. In this paper, the work intensity variables are set in accordance with the following two dimensions: on the one hand, persons’ weekly working hours are obtained as per the questionnaire “the number of hours worked this week”; and on the other hand, the 44 h of working hours per week which is stipulated by the Chinese Labor Law is adopted as the standard to determine the work intensity. Furthermore, the work intensity variable is assigned a value of 1 when the interviewee’s weekly working hours exceeded 44 h, otherwise, 0 when the stated working hours are less than or equal to 44 h. In addition, the heterogeneity analysis of work intensity results put forward that the promotion effect of access to public health education services in urban areas on their fertility intention is weakened as there is an increase in the work intensity of the rural migrant population. This result is in line with expectations, therefore this paper confidently argues that the size of work intensity is a crucial influencing factor in the opportunity and willingness of the rural migrant population to obtain education services in urban public health. On the one hand, individuals with high work intensity may show more willingness to involve in leisure and recreational programs to alleviate work fatigue during their spare time; thus, resulting in a relatively lower willingness to receive health education services in urban public health; on the other hand, urban public health education services are majorly conducted through lectures, consultation, SMS, and propaganda, among which lectures and consultation are specifically limited by time, and persons with high work intensity tend to miss service hours due to work schedule.

**Table 8:** Analysis of heterogeneity—group differences

Variables	(1)	(2)	(3)
	Intensity work	Intention to stay	Education level
UPHES	0.133***	0.044	0.126***
	(0.030)	(0.036)	(0.033)
UPHES_IW	− 0.111***		
	(0.023)		
UPHES_IS		0.101***	
		(0.030)	
UPHES_EL			− 0.166***
			(0.047)
Control variable	Yes	Yes	Yes
Provincial effect	Yes	Yes	Yes
Constant	1.637***	1.657***	1.557***
	(0.195)	(0.196)	(0.196)
Observations	90,279	90,279	90,279

*** *P* < 0.01, ** *P* < 0.05, * *P* < 0.1

Table 8 (2) populates the results of the heterogeneity analysis of intention to stay. Meanwhile, the variable for intention to stay is set based on the questionnaire question, “Do you intend to stay in the future?” Accordingly, the variable of intention to stay is assigned to 1 when the interviewee responds “yes”, otherwise when the interviewee responds “no”, the variable of intention to stay is assigned to 0. Besides this, the UPHES_IS is defined as the interaction item between education service in urban public health and the work intensity variable. The results of the heterogeneity analysis confirm that access to urban public health education services is more effective in enhancing fertility intentions for those who are willing to stay in the area than those who are not willing to stay. Thus, it is logical to infer that the stronger willingness of rural migrant people to stay in the city often implies a higher degree of recognition, adaptation, and integration in the new environment, and a higher willingness to recognize the quality of public services in the surrounding environment. Therefore, the willingness to accept and demand urban public health education services is relatively higher among rural migrants who show more willingness to stay in urban regions.

Table 8 (3) presents the results of heterogeneity analysis in terms of the education level. Notably, the education level variable is set consistent with the interviewees’ education level. Owing to China’s “9-year compulsory education”, the variable is assigned to 1 when the interviewee reports a high school education or above; conversely, the education level variable is assigned to 0 when the education level of the interviewee is below high school. Similarly, the UPHES_EL is described as UPHes_EL for interaction between education service in urban public health and work intensity variable. Parallel to this, the results of the heterogeneity analysis of education level depict that rural migrants’ access to urban public health education services becomes less effective in promoting their fertility intentions, with the increase in the education level of rural migrants. Since the more educated rural migrants are, the more self-learning ability these persons demonstrate, the more channels these migrants have to acquire health knowledge, and the less these rural migrants rely on education services in urban public health.

## Discussion

This paper focuses on the influences of public health education services in urban areas on the fertility intentions of rural migrant persons. Consequently, there exist certain differences between this study and the extant relevant literature in terms of methodology, research content, and study findings. In this paper, the study discussion is focused on the above three dimensions.

Discussion on the research subjects. While the extant literature has investigated the matter of public services or social benefits associated with fertility intentions majorly from the perspective of social security [9, 15], pensions [8, 35], and child allowances [33], this research article examines the influence mechanisms of public services on fertility intentions in the context of urban public health education services. Simultaneously, this research paper focuses on the rural migrant population, which encounters more pressure from economic, social, cultural, life, and health aspects as compared to the local non-migrant population. In addition to this, there exists an unequal supply of public health services, which makes the rural floating population warrant more factors from health and medical treatment when making reproductive decisions. As a result, the research object of this article is more aligned and fills a gap in the present literature, which is of vital practical significance for not only integrating rural migrants into urban life but alleviating their fertility pressure.

Discussion on the research methodology. This research article somewhat differs from the previous studies in terms of the research methodology. The possible selectivity bias of the problem under study has been taken into account when performing the sample processing, therefore, the existing literature has been processed majorly through the PSM approach [47]. Further, this paper adopts the entropic balanced matching method in order to avoid the significant drawbacks of poor covariate balance and low accuracy of matching scores when dealing with high-dimensional data. The proposed method exhibits the benefits of being more balanced, flexible, and robust than PSM, to weaken the selectivity bias. In terms of coping with the endogeneity issue, the instrumental variable method is chosen in the study paper for validation, in order to address potential endogeneity concerns such as reciprocal causality and omitted variables. From the perspective of instrumental variables selection, the local anti-corruption efforts are innovatively used as the instrumental variable of public health education services, which better weakened the potential endogeneity concern associated with this paper; thereby, obtaining the net influence of public health education services on fertility intention of rural migrant population, and eventually enhancing the credibility of the study conclusions.

Discussion on the research findings. The major ideas presented in this paper are largely similar to those proposed by Chen and Liu [7], Wang and Peng [42], Yakita [44], and Rupa [38]. However, some scholars have reached seemingly opposite consensus in the context of pension pressure. These scholars believe that the optimization and improvement in public services shall not only lower the pension pressure but also weaken the role of “child-rearing”, thus, leading to a decline in fertility [3, 9, 10]. Consequently, this research supports the viewpoint that the association between social security and fertility intentions is affected by factors such as social security capacity and household economic level. Therefore, there are two major reasons behind the differences between this paper and related research studies: On the one hand, the mean level of household economy and overall social security capacity in China is relatively low. Further, from the Chinese experience, the level of social security is still in the promotion stage for increasing fertility intention. On the other hand, there exists a difference in the mechanism of action between health education services and general social security on the rural migrants’ fertility intention, with the former acting more directly on the recipients themselves (such as physical quality and mental health) whereas the latter impacting more on family economic burden and pension pressure, therefore there exists a positive association between health education services and fertility intention.

## Conclusions and policy recommendations

### Conclusion

This research paper anticipates the influence of education services in urban public health on the fertility intentions of rural mobile populations with the help of 2018 CMDS data. As a result, this study draws the following conclusions: (i) Firstly, urban public health education services effectively promote the fertility intentions of the rural migrant population. In addition, the proposed conclusion still holds after the robustness test using entropic equilibrium matching, replacement empirical models, and instrumental variable methods; (ii) Secondly, this article further verifies that improvement in the health status serves as an important mechanism by which urban public health education services affect the fertility intentions of the rural migrant population; (iii) Thirdly, urban public health education services employing public health consultation, SMS, face-to-face consultation, and promotional materials effectively enhances the fertility intentions of the rural migrant population; (iv) Fourthly, there is a significant effect of urban public health education services on improving the fertility intentions of the rural migrant population with high willingness to stay and low work intensity, whereas the effect of education level is relatively insignificant. As a result, these implications not only enrich the theories associated with fertility intentions but also extend new insights into optimizing the mode of education services in urban public health development.

### Policy recommendations

Based on the aforementioned findings, this paper puts forward several policy recommendations in the following fields:

1. Improvement in the quantity and quality of public health service supply. Public health services comprise an important program to improve health, relieve the routine stress of life and support the living standard of the population. Since the introduction of the public health service program in 2009, there has been a significant improvement in the public health services of China. Although, the supply still exceeds the demand, when compared to the size of China’s large population. Parallel to this, most of the present mobile population does not have an in-depth understanding of public health services and lacks the awareness to actively accept public health services actively. Thus, in order to address the aforementioned problems related to public health service supply and achieve a balanced and steady improvement in the living standards of urban and rural residents, there is a need to integrate the following recommendations. Firstly, there is a significant need to further enhance service supply, improve the quantity of public health service supply, increase the financial investment in public health services, specifically in health education, and reduce the pressure on the rural migrant population on childbirth. Secondly, the present situation also warrants strengthening the training of public health service personnel, establishing their positive and patient service consciousness, improving their professional skills, and further optimizing the quality of public health services. Finally, the reputation should also be enhanced to support more mobile populations to understand public health services in order to increase their awareness of education services in urban public health.
2. Enriching the ways and connotations of urban health education services. This study reports significant differences in terms of the effectiveness of receiving urban public health education services in promoting fertility intentions under different delivery modes and rural migrant population groups of urban public health education services. Firstly, there is a need to adjust the approach to health education, in order to make it more flexible. On the one hand, the execution of health education based on the development background of the present era and residents’ daily habits adjusts or reduces the traditional publicity methods since residents pay little attention (such as publicity and columns). On the other hand, targeted and efficient health education services must be supported in appropriate manners such as face-to-face consultation and personalized mode. Secondly, there is a need to further expand the health education model. Although health education in the shape of health knowledge lectures deepens the content of health education, the knowledge spillover effect is not appropriate. Further, the development mode and connotation of health education must be enriched through more exciting and vivid forms including community health-knowledge competitions, in order to enhance the willingness of residents to participate. Finally, a service model should be built based on the crowd precision docking by performing an excellent job in the questionnaire survey of residents' health education, classifying the educated population in accordance with their health needs, closely focusing on residents’ expectations and demands for health education, and offering complementary health education services to the residents.
3. Guarantee the development of equalizing public health services for the mobile population. On the one hand, there is a need to further improve the top-level development design of public services equalization. Consequently, the public health services for the mobile population must be incorporated into the social and economic development planning of each region; while formulating a complete public health service system for the mobile population. Simultaneously, a scientific and practical collaborative governance mechanism needs to be constructed to promote the equalized development of public health services for the mobile population. On the other hand, there is a substantial need to optimize the transfer mechanism of regional household registration, reduce the difficulty for rural migrants to receive public health services, lower the missed opportunities for rural migrants to receive health education services in towns due to household registration transfer problems, and improve the convenience of community public health services.

## Data Availability

The datasets generated during and/or analyzed during the current study are available from the corresponding author on reasonable request.
